# Unraveling Anaerobic Metabolisms in a Hypersaline Sediment

**DOI:** 10.3389/fmicb.2022.811432

**Published:** 2022-03-16

**Authors:** Juan Ignacio Solchaga, Juan Pablo Busalmen, Débora Nercessian

**Affiliations:** ^1^Instituto de Investigaciones Biológicas, Universidad Nacional de Mar del Plata – CONICET, Mar del Plata, Argentina; ^2^Laboratorio de Bioelectroquímica, INTEMA - CONICET, Universidad Nacional de Mar del Plata, Mar del Plata, Argentina

**Keywords:** hypersaline sediment, anaerobic microorganisms, electron acceptor, electrode respiration, anaerobic diversity

## Abstract

The knowledge on the microbial diversity inhabiting hypersaline sediments is still limited. In particular, existing data about anaerobic hypersaline archaea and bacteria are scarce and refer to a limited number of genera. The approach to obtain existing information has been almost exclusively attempting to grow every organism in axenic culture on the selected electron acceptor with a variety of electron donors. Here, a different approach has been used to interrogate the microbial community of submerged hypersaline sediment of Salitral Negro, Argentina, aiming at enriching consortia performing anaerobic respiration of different electron acceptor compounds, in which ecological associations can maximize the possibilities of successful growth. Growth of consortia was demonstrated on all offered electron acceptors, including fumarate, nitrate, sulfate, thiosulfate, dimethyl sulfoxide, and a polarized electrode. *Halorubrum* and *Haloarcula* representatives are here shown for the first time growing on lactate, using fumarate or a polarized electrode as the electron acceptor; in addition, they are shown also growing in sulfate-reducing consortia. *Halorubrum* representatives are for the first time shown to be growing in nitrate-reducing consortia, probably thanks to reduction of N_2_O produced by other consortium members. Fumarate respiration is indeed shown for the first time supporting growth of *Halanaeroarchaeum* and *Halorhabdus* belonging to the archaea, as well as growth of *Halanaerobium*, *Halanaerobaculum*, *Sporohalobacter*, and *Acetohalobium* belonging to the bacteria. Finally, evidence is presented suggesting growth of nanohaloarchaea in anaerobic conditions.

## Introduction

Hypersaline environments such as saline lakes are inhabited by halophilic microorganisms that, thanks to a “salt-in” strategy or the production and/or accumulation of organic osmolytes, are able to maintain the cell cytoplasm isosmotic with the environment for growing optimally at salt concentrations ranging from 150 to 300 gL^–1^ (2.5–5.2 M) NaCl (Oren, 1999). Maintaining osmolarity implies an extra energy cost that strongly conditions diversity in these environments. The aquatic counterpart of the microflora in hypersaline systems has been thoughtfully studied and is typically dominated by aerobic archaea and bacteria, some of which can also live as facultative anaerobes (Oren and Trüper, 1990; Oren, 1991; Bonete et al., 2008; Antunes et al., 2011). On the other hand, the number of studies on the microflora of the more reduced submerged hypersaline sediments is very limited (Kim et al., 2012; Sirisena et al., 2018; Vera-Gargallo and Ventosa, 2018; Martínez et al., 2021), and there is a marked lack of information on the anaerobic representatives of hyperhalophiles. The energy cost of thriving in high salt concentration may be presumed as unaffordable for anaerobic metabolisms (Oren, 2011); nevertheless, information emerging from molecular ecology studies based on the small subunit rRNA gene phylogeny progressively demonstrates the existence of diverse anaerobic communities in submerged sediments (Jiang et al., 2006; Vavourakis et al., 2018) in which most members remain uncultured.

This work focuses on microorganisms that perform anaerobic respiration in the community of submerged hypersaline sediment of Salitral Negro, Argentina. Underlying the saline crust and up to 1 m deep, the sediment at this site is composed of very fine friable layers of sodium chloride and sulfate, alternating with dark muds, composed of sand and clay with intercalations of gypsum and sulfate minerals such as glauberite [Na_2_Ca(SO_4_)_2_], anhydrite (CaSO_4_), and astrakanite [Na_2_Mg(SO_4_)_2_⋅4H_2_O]. Salt minerals represent up to 95% of the sediment. The chemical analysis of the brines indicates the presence of Na^+^ (up to 95 g/L), Mg^2+^ (up to 34 g/L), and K^+^ (up to 7.4 g/L) within main cations and Cl^–^ (up to 171 g/L) and SO_4_^–2^ (up to 89 g/L) within main anions (Cangioli, 1971; Pires, 2017).

Upon sequential enrichment in defined culture media containing non-fermentable carbon substrates as electron donors and different compounds as electron acceptors, several consortia were obtained that contained archaea and bacteria that respire nitrate, sulfate, thiosulfate, dimethyl sulfoxide (DMSO), or a polarized graphite electrode. Kinetics of community growth on every oxidizer was measured, and the composition of the resulting consortia was determined by denaturing gradient gel electrophoresis (DGGE), followed by the 16S rRNA gene sequence analysis. Finally, the composition of the enriched consortia was examined relative to the use of electron donors and acceptors and ecological interactions.

## Materials and Methods

### Culture of Microorganisms

The sample of anaerobic sediment was collected in a sterile container from Salitral Negro saltern, located in La Pampa province, Argentina (38°43′01′′ S, 64°09′01′′ W) on January 24, 2018, and transported to the laboratory in a hermetic box. Aliquots of 5 g were used to inoculate hermetic 50-mL sterile vials containing 40 mL of a defined medium on buffer PIPES 25 mM (pH 6.8) containing (g/L): NH_4_Cl, 0.5; KCl, 5; NaCl, 195; CaCl_2_⋅2H_2_O, 0.95; MgCl_2_⋅6H_2_O, 40.7; NaBr and 0.65; KH_2_PO_4_, 0.2; medium was supplemented with vitamins and trace minerals according to nitrilotriacetic acid 15; MnSO_4_⋅H_2_O 5; FeSO_4_⋅7H_2_O 1; CoCl_2_⋅6H_2_O 1; ZnCl 1.3; CuSO_4_⋅5H_2_O 0.1; AlK(SO_4_)_2_⋅12H_2_O 0.1; H_3_BO_3_ 0.1, Na_2_MoO_4_ 0.25; NiCl_2_⋅6H_2_O 0.24; Na_2_WO_4_⋅2H_2_O 0.25; and vitamins (mgL^–1^): biotin 0.02, folic acid 0.02, pyridoxine.H_2_O 0.1, riboflavin 0.0005, thiamine 0.0005, nicotinic acid 0.0005, pantothenic acid 0.0005, cyanocobalamin 1 × 10^–5^, *p*-aminobenzoic acid 0.0005, and thioctic acid 0.0005 (Balch et al., 1979). Fumarate (50 mM) was added as electron acceptor, and lactate or acetate (20 mM) was used as the carbon source. Vials were deoxygenated by bubbling sterile N_2_ for 10 min before use. Cultures were developed in the dark at 42°C for 1 month. Three successive replicates were performed to obtain a stable consortium. Growth was followed by measuring optical density at 600 nm (OD_600_) in a UNICO 2800 UV/VIS spectrophotometer.

Lactate-oxidizing consortia were used as the inoculum to test alternative electron acceptors; 1 mL of lactate-oxidizing culture was inoculated in vials containing 50 mL of the defined medium and one of the following electron acceptors: fumarate (50 mM), NO_3_^–^ (30 mM), DMSO (30 mM), thiosulfate (30 mM), and sulfate (30 mM). Also, a polarized electrode was used as an electron acceptor (see below). Lactate 20 mM was the carbon source. Cultures were incubated in the dark at 42°C until the stationary phase.

### Electrochemical Assays

For electrochemical growth, 10 mL of the same lactate-oxidizing consortia was inoculated in a single-compartment three-electrode electrochemical reactor containing 90 mL of deoxygenated defined medium. The working electrode was a 0.4-cm-diameter graphite bar (XTG-15, Carbograf, Argentina) polarized to 0.2 V against an Ag/AgCl–3 M NaCl reference electrode (+0.202 V vs. the standard hydrogen electrode [SHE]); in the absence of any soluble electron acceptor, it functioned as the only respiratory option supporting growth. The surface of the working electrode was renewed before each experiment by polishing to grade 1,000 with carbon paper, sonicating by three pulses of 5 s in distilled water to remove debris and washing with deionized water. A 15-cm-long platinum wire was used as the counterelectrode. Triplicate reactors were run in batch for 48 h, to be later connected to a continuous medium supply, at a dilution rate of 0.02 h^–1^. Reactors were continuously bubbled with N_2_ gas to ensure anaerobic conditions. All the experiments were performed under permanent magnetic stirring.

The electrochemical experiments were performed using an Autolab PGSTAT101 potentiostat controlled by the NOVA 1.11 software. Chronoamperometry was performed at the indicated potential along the necessary time to reach stationary current, noting that current was taken here as an estimation of growth (Schrott et al., 2014). For cyclic voltammetry, the potential was scanned between 0.4 and −0.8 V starting anodically from the potential used for growth. The scan rate was 0.01 Vs^–1^. All potentials are expressed against the SHE unless indicated.

### DNA Extraction, Polymerase Chain Reaction, and Denaturing Gradient Gel Electrophoresis Analysis of SSU rRNA Genes

DNA was extracted from 1 mL of every culture when it reached the stationary phase using an UltraClean Microbial DNA isolation Kit MO Bio. Conserved regions of the prokaryotic 16S rRNA genes were amplified by polymerase chain reaction (PCR) using bacteria: 341f-GC (5′-CGC CCG CCG CGC GCG GCG GGC GGG GCG GGG GCA CGG GGG GCC TAC GGG AGG CAG CAG-3′) and archaea: 344f-GC (5′-CGC CCG CCG CGC CCC GCG CCC GTC CCG CCG CCC CCG CCC GAC GGG GYG CAG CAG GCG CGA-3′) targeting primers as previously described (Amann et al., 1990; Muyzer et al., 1993). The PCR products were purified with the DNA PuriPrep-GP Kit (Inbio-HighWay) according to the manufacturer’s recommendations. Five hundred nanograms of each purified PCR product was then loaded in acrylamide denaturing gradient gels (0.75 mm thick). DGGE was performed in 1 × Tris–acetic acid–EDTA buffer (40 mM Tris, pH 8.0; 20 mM acetic acid; 1 mM EDTA) at 60°C under 60-V polarization for 16 h. The denaturing gradient was 45–65% for *Archaea* and *Bacteria.* After running, DGGE gels were stained for 30 min with Sybr Gold and visualized under blue light in a transilluminator. Selected DNA bands were excised from the gels, resuspended in 20 μL of sterile distilled water and incubated at 4°C for 16 h. DNA from each band was then amplified again by PCR using the same primers. Obtained PCR products were sequenced using Standard Seq Service from Macrogen (Korea). The BLASTn tool at the National Center of Biotechnology Information website^1^ was used to find the closest sequences in the Nucleotide Collection. The DGGE band sequences obtained were deposited in the GenBank or NCBI Short Read Archive databases. The composition of enriched communities was analyzed in relation to electron donor and acceptors utilization, by considering available information on the closest known relatives for every organism and genetic information taken from the Kyoto Encyclopedia of Genes and Genomes (KEGG) database^2^.

## Results and Discussion

### Enrichment of Anaerobic Respirers

Fumarate reduction has been reported in halophilic archaea and bacteria (Thauer et al., 1977; Oren, 1991, 2016) and is considered as the most widespread anaerobic respiration among microorganisms (Kröger et al., 1992). It was used here as a first step to select a stable consortium of anaerobic respirers from the sediment community taken from the Salitral Negro. Selective cultures were developed using fumarate as a general purpose electron acceptor and acetate or lactate as the electron donor. In addition, and taking into account that lactate can be fermented (Alexander and Davies, 1963; Prins et al., 1975; Tang et al., 1989), cultures on lactate were developed also in the absence of fumarate to evaluate the extent of fermentative growth.

Cultures grown on acetate rapidly developed to enter into stationary growth after approximately 10 days, reaching a final OD_600_ of 0.4. Those developed on lactate, on the other hand, grew exponentially to reach a maximal OD_600_ of 0.8 after approximately 20 days (Supplementary Figure 1A). To gain information about biological diversity in the selected communities, DGGE of the 16S rRNA gene PCR products was performed, using degenerate primers for archaea and bacteria. Typical results are presented in Supplementary Figure 1B to illustrate the detection of at least 9 different archaea and 17 different bacteria. Only three of the detected archaea developed in this case on both acetate and lactate; another three developed only on acetate, and the last three only on lactate. Within bacteria, only two were able to grow on both substrates, whereas five were found growing only on acetate and 10 on the more reduced substrate, lactate. Along replicate experiments, diversity was always greater on lactate, in agreement with the higher potential difference between this electron donor and fumarate, as compared with that between the couple acetate/fumarate (Thauer et al., 1977). Growth was not detected on lactate in the absence of fumarate, confirming the absence of lactate fermentation in the sediment community.

### Growth on Different Electron Acceptors

As lactate-oxidizing consortia accumulated much more biomass (Supplementary Figure 1A) and presented always a greater diversity than those grown on acetate, they were selected as the source to inoculate selective culture media containing lactate and different oxidizers, in order to explore respiratory variants in the sediment microflora. Selected electron acceptors covered a wide range of redox potentials: nitrate = 0.433 V, DMSO = 0.160 V, fumarate = 0.033 V, polarized electrode = 0.00–0.20 V, thiosulfate = −0.189 V, sulfate = −0.217 V (Thauer et al., 1977; Cord-Ruwisch et al., 1988; Wissenbach et al., 1990).

**FIGURE 1:**
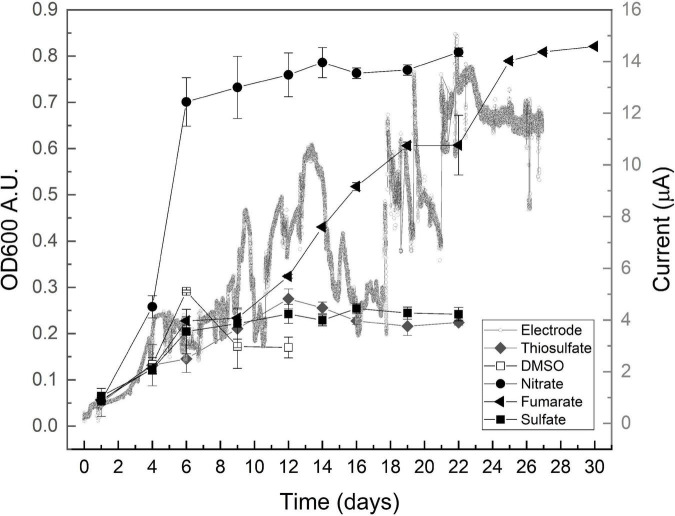
Growth profiles obtained on lactate and the indicated electron acceptors after inoculating with consortium enriched on lactate/fumarate couple. OD_600_ data are presented as media ± standard deviation (*n* ≥ 3). A typical chronoamperometry curve is presented.

Growth curves are shown in Figure 1, and growth parameters derived from these curves are listed in Table 1, together with the redox potential of electron acceptors. As shown by the results in Figure 1, all assayed oxidizers supported growth to some extent, evidencing versatility of the community for anaerobic respiration. Nitrate availability is known to below the sampled site (Pires, 2017) as typically found in saline environments (Mancinelli and Hochstein, 1986), but cultures grown on nitrate were here the most successful by developing a maximal OD_600_ of approximately 0.8, after growing exponentially during 4 days at a doubling time of 1.35 days (Table 1). In comparison, those grown on fumarate reached similar optical density values, but exhibited a much larger doubling time of 4.9 days (Table 1).

**TABLE 1:** Doubling time and maximal OD_600_ for communities growing on lactate and different electron acceptors obtained from data in Figure 1.

Electron acceptor	E_0_’ (V vs. SHE)	Doubling time (days)	OD600_max_
Nitrate	0.433^a^	1.36	0.8
DMSO	0.160^b^	2.17	0.3
Fumarate	0.033^a^	4.88	0.8
Electrode	0.00–0.20	6.12	ND
Thiosulfate	−0.189^c^	3.06	0.25
Sulfate	−0.217^c^	3.03	0.23

*Redox potentials of electron acceptors obtained from literature are included. The electrode potential was derived from supplementary electrochemical data as explained in the text.*

*^a^Thauer et al., 1977.*

*^b^Wissenbach et al., 1990.*

*^c^Cord-Ruwisch et al., 1988.*

Dimethyl sulfoxide is often found in saline environments as the resulting product of the oxidation of dimethyl sulfide (Oren and Trüper, 1990; Sorokin et al., 2017) and is known to be used by archaea and bacteria as an electron acceptor for anaerobic respiration. Growth on DMSO was here at a doubling time of 2.17 days (Table 1) (faster than that on fumarate in the same medium) but reached a maximal OD_600_ of approximately 0.3 to later stabilize at a much lower value of 0.15 (Figure 1), thus exhibiting a relatively poor performance in supporting growth. Sulfate and thiosulfate are known to support the growth of several halophiles in anaerobic environments that indeed influence relevant geochemical cycles (Warthmann et al., 2005; Vavourakis et al., 2018). Cultures grown on sulfate and thiosulfate also showed a low performance here, reaching a maximal OD_600_ of approximately 0.25 despite duplicating relatively faster than those growing on fumarate, at doubling times of approximately 3 days (Table 1).

Microorganisms interacting with electrodes are presently boosting a myriad of biotechnological processes (Clauwaert et al., 2008; Rosenbaum and Franks, 2014; Rodrigo Quejigo et al., 2019; Prévoteau et al., 2020), but little is known yet about halophiles with this capacity (Carmona-Martínez et al., 2013; Shrestha et al., 2018). Although not directly comparable in terms of growth yield, data from cultures grown here by using a polarized electrode as the electron acceptor show that they developed in the same time frame of those growing on fumarate, exhibiting a doubling time of approximately 6 days and reaching the maximal current beyond day 12 (Figure 1 and Table 1). Owing to the fact that no other electron acceptor was provided in the culture media and recalling the absence of fermentative metabolism, these results confirm that exocellular respiration by interaction to a polarized electrode is possible within anaerobic hyperhalophiles, a topic that remained virtually unexplored until present days. Upon performing a voltammetric analysis on the electrode, a signal characterized by a reduction wave peaking below −0.1 V, accompanied by an oxidation wave peaking at 0.2 V, was obtained (Supplementary Figure 2) that is presumed to correspond to the electrochemical redox response of the molecule in the controlling step of the electron transport pathway conducting respiratory electrons to the cell exterior. The process exhibited an estimated half wave potential of 0.0 V (Supplementary Figure 2). According to these results, for the material to act as an electron acceptor, the effective electrode potential may be in the range of 0.0–0.2 V (Table 1 and Supplementary Figure 2).

As shown by data compiled in Table 1, the mean doubling time of the obtained communities progressively increased as the potential of the electron acceptor decreased, except for fumarate and electrode that sustained a relatively low growth rate despite having positive potentials. Maximal OD_600_ reached by growing on every acceptor was, on the other hand, directly related to the acceptor potential. Again, the exception was fumarate that sustained growth to an OD_600_ as high as that measured on nitrate (Table 1). Growth on electrodes, on the other hand, did not progress as planktonic growth as OD_600_ virtually did not change (data not shown) despite the observed current increment (Figure 1). This points to the development of an electrode-associated community as typically observed in electrode-reducing bacteria (Robuschi et al., 2013; Schrott et al., 2014), which warrants future research to explore growth of hyperhalophilic electroactive biofilms.

**FIGURE 2:**
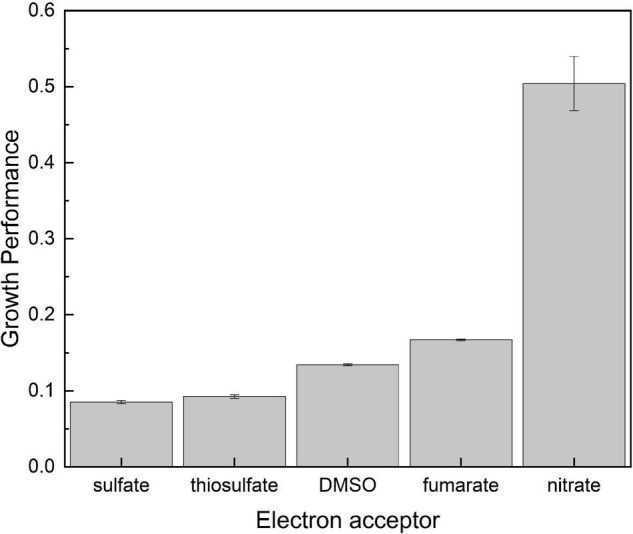
Growth performance calculated as OD_600max_/doubling time as related to the available electron acceptor on which consortia were developed.

To have a much clearer picture on the dependence of growth on the electron acceptor potential, the two growth-related parameters were combined to calculate growth performance (GP) as follows: GP = OD_600*max*_/doubling time. Results are shown in Figure 2. As expected, lowest GP values were obtained on sulfate and thiosulfate, which ranged below 0.1 (Figure 2). Fumarate and DMSO, on the other hand, presented intermediate values in consequence of the large biomass development supported by the former and the short doubling time observed on the latter. Finally, cultures grown on nitrate clearly outperformed all the others exhibiting a GP as high as 0.5 in consequence of the very short doubling time and the high final optical density they showed.

It is widely accepted that growth strongly depends on the energy available to synthesize ATP, which can be calculated from the potential difference between electron donating and accepting reactions (Thauer et al., 1977). Assuming a two-electron transferring mechanism (*n* = 2), a potential difference (ΔE′) of approximately 250 mV is required to allow the synthesis of 1 mol of ATP from ADP and Pi (ΔG′ = −nFΔE′ = 2 × 23.06 × 1.04 kJ/mol = −48 kJ/mol). Listed in Table 2 are global reactions and calculated ATP for the oxidation of lactate with every oxidizer used in this work.

**TABLE 2:** Energy available for growth and amount of ATP that can be produced upon coupling lactate oxidation to utilization of the indicated electron acceptors.

Electron acceptor	Redox reaction	ΔG° (KJ)	ATP produced
Nitrate	Lactate + 2NO_3_^–^ + H^+^ → acetate + HCO_3_^–^ + 2H_2_ + 2NO_2_^–^	−322.2	7.7
DMSO	Lactate + 2C_2_H_6_SO + H ^+^ → acetate + HCO_3_^–^ + 2H_2_ + 2C_2_H_6_S	−221.5	5.3
Fumarate	Lactate + 2H_2_O + 2C_4_H_4_O_4_ + 2H_2_ → acetate + HCO_3_^–^ + H^+^ + 2H_2_ + 2C_4_H_6_O_4_^–2^	−186.6	4.5
Thiosulfate	Lactate + S_2_O_3_^–2^ + 2H_2_ → acetate + HCO_3_^–^ + H^+^ + 2HS^–^ + H_2_O	−176.3	4.2
Sulfate	2 Lactate + SO_4_^2–^ → 2 acetate + 2HCO_3_^–^ + HS^–^ + H^+^	−156.1	3.7
Electrode	Lactate + 2H_2_O + NAD^+^ → NADH + acetate + HCO_3_^–^ + 2H_2_	−100.3	2.4

*Listed are global reactions, and ATP yields calculated on the base of potential Gibbs free energies of formation reported by Thauer et al. (1977) for the oxidation of lactate with every oxidizer.*

Data in Table 2 show that nitrate reduction yields between 32 and 50% more energy than the other oxidizers in agreement with the higher GP shown in Figure 2. Respiration of DMSO on the other side supported a lower GP (Figure 2) despite yielding gretaer than 15% more energy than fumarate (Table 2). Energetic yield of respiring fumarate is indeed nearly the same obtained from thiosulfate reduction, but communities growing on fumarate presented a higher performance reaching a much higher OD_600_ in the long term (Figure 2 and Table 1). Regarding electrochemical growth, cyclic voltammetry indicates an efficient electric communication between cells and the electrode (Supplementary Figure 2), but results in Table 2 show that the amount of energy obtained through electrode respiration is quite lower than that obtained from the other oxidizers, in part explaining why growth was very limited in the electrochemical reactors and was even below the quantity required for biomass extraction from electrodes and quantification. Besides the influence of thermodynamics, growth variables strongly depend on intrinsic properties of every organism as elegantly shown by Robinson et al. (2005). In this direction, it has to be taken into account that results presented in Figures 1, 2 and in Table 1 correspond to the response of a complex community in which every member may have a different growth behavior in amplitude and time, depending indeed on putative ecological interactions between them and the chemical changes every one may introduce to the system. In the particular case of growth on electrodes, reasons may include the imperfect electric communication in between cells (Ordóñez et al., 2016) and the impossibility to form conductive biofilms, which thus may limit growth to a monolayer of cells attached to the electrode. All these considerations may partially explain the lack of correlation between energetic considerations (Table 2) and actual growth of consortia (Figure 1 and Table 1).

### Diversity of Communities Enriched on the Different Oxidizers

All sulfate-reducing bacteria and also most fumarate-reducing bacteria are strict anaerobes, but they frequently share the ability to respire various electron acceptors. Sulfate-reducing bacteria can frequently use fumarate, whereas fumarate-reducing bacteria can also use either sulfate or nitrate; nitrate-reducing bacteria can also use either fumarate or nitrite, being indeed facultative anaerobes as they can also use oxygen as electron acceptor (Thauer et al., 1977). The same is known to occur within haloarchaea. While *Halobacterium salinarum* has been shown to use fumarate, DMSO, and trimethylamine N-oxide (TMAO) as electron acceptors, *Haloferax denitrificans* can use fumarate, TMAO, and nitrate, and *Haloarcula marismortui* can use DMSO, TMAO, and nitrate (Oren, 1991; Torregrosa-Crespo et al., 2020).

As previously done with acetate/fumarate- and lactate/fumarate-growing consortia, DNA was extracted from cultures grown on lactate and the different oxidizers. The 16S rRNA gene was amplified using degenerate primers for bacteria and archaea, and the PCR products were analyzed by DGGE. Typical results obtained from the DGGE analysis are presented in Supplementary Figure 3. As can be observed in this example, some bands were found to be present in more than one lane, indicating the occurrence of strains that respire more than one oxidizer. This point is analyzed in depth later.

Bands detected on genetic analysis of all enriched consortia were excised for amplification and sequencing. DNA amplification of bacterial bands was successful on samples from cultures grown on nitrate, fumarate, thiosulfate, sulfate, and DMSO, but failed in the case of cultures grown on electrodes. Within archaea bands, successful DNA amplification was on samples from cultures grown on nitrate, fumarate, sulfate, and electrode and failed in the cases of thiosulfate and DMSO.

The products of successful amplifications were sequenced and compared with NCBI genetic data base using BLAST public tool to find the closest sequences. The results are summarized in Table 3, where the affiliation of every band is presented as related to the reducer and the oxidizer on which the organism was enriched. A total of 20 different archaeal sequences could be obtained, from which 9 were identified as corresponding to previously uncultured archaea and 11 were affiliated to already known organisms (Table 3). In the case of unknown organisms, the closest type strain is indicated in Table 3 for reference. Within bacteria, a total of seven different sequences were obtained, from which three matched with previously uncultured organisms and four to already known bacteria. Archaeal diversity was dominated by organisms that have *Halorubrum* and *Haloarcula* as the closest related known genera, represented by six and seven different sequences (Table 3, Hrr.:A5, A6, A8, A9, A11, and A17; Har.: A7, A10, A13, A14, A15, A16, and A19), respectively. Three other sequences (A1, A2, and A3) have a member of the *Nanohaloarcheota* phylum as the closest known organism (Table 3), whereas other two sequences have *Halanaeroarchaeum sulfurireducens* strain HSR2 (A18, 97.42%) and an uncultured *Halorhabdus* sp. clone A2_31 (A20, 94.44%) as the closest known organism, respectively. It is to note at this time that sequence A4 amplified by using archaea-directed primers matched with *Sporohalobacter lortetii* strain CEJFYE2D with a 98% identity. Although not frequently, this cross-domain recognition is known to occur. *S. lortetii* CEJFYE2D was also the closest match of band B6 with a 96% identity, again indicating the presence of members of this genus in the enriched consortium. Finally, bacterial diversity also included sequences with *Halanaerobium saccharolyticum* (B7, 100%; B4, 99%; and B1, 97.98%), *Halanaerobaculum tunisiense* 6SANG (B3, 96%), and *Acetohalobium arabaticum* DSM 5501 (B5, 97.70%), as the closest known organisms.

**TABLE 3A:** 16S rRNA gene sequences detected in cultures developed on acetate or lactate as the electron donor and the indicated electron acceptors upon amplification, DGGE separation, reamplification, and sequencing of archaeal DNA.

Operational Taxonomic Unit	Accession no.	Closest match (identity%)—accession no.	Acetate	Lactate					
				
		Closest identified type strain (identity%)—accession no.	Fumarate	SO_4_^–2^	S_2_O_3_^–2^	Fumarate	DMSO	Electrode	NO_3_^–^
A1	SAMN22936717	Uncultured archaeon clone BOX1_E8 (96%)—KU196432.1	**+**			**+**			
		Uncultured *Nanohaloarchaeota* (89.46%)—KU760777.1 Nanohaloarchaea archaeon SG9, complete genome (88%)(CP012986.1)							
A2	SAMN22929635	Uncultured archaeon clone BOX1_E8 (98%)—KU196432.1	**+**						
		Uncultured *Nanohaloarchaeota* (93.62%)—KU760777.1 Nanohaloarchaea archaeon SG9, complete genome (88%)(CP012986.1)							
A3	SAMN22936718	Uncultured archaeon clone BOX1_E8 (96%)—KU196432.1	**+**						
		Uncultured *Nanohaloarchaeota* archaeon (91.82%)—KU760777.1 Nanohaloarchaea archaeon SG9, complete genome (88%)(CP012986.1)							
A4	Z314654	Uncultured *Halobacteroidaceae* (99%)—DQ386210.1	**+**			**+**			
		*Sporohalobacter lortetii* strain CEJFYE2D (98%)—KU180227.1							
A5	MZ314655	*Halorubrum* sp. AJ102 isolate AJ102 (99%)—HE802582.1				**+**			
A6	MZ314656	*Halorubrum saccharovorum* strain MLR66 (94%)—KY411787.1		**+**				**+**	**+**
A7	MZ314657	*Haloarcula* sp. TG5 16S (99%)—KU051673.1							**+**
A8	OL362290	Uncultured haloarchaeon clone XKL38 (95.83%)—JN714435.1		**+**					**+**
		*Halorubrum* sp. strain G16-1 (95%)(MH106556.1)							
A9	OL362291	*Halorubrum saccharovorum* strain MLR66 (94%)—KY411787.1		**+**				**+**	**+**
A10	MZ314658	*Haloarcula* sp. strain BT1 (99.76%)—MN134066.1						**+**	
A11	MZ314659	*Halorubrum* sp. strain G16-1 (96.12%)—MH106556.1				**+**		**+**	
A12	SAMN22929636 (NCBI)	Uncultured archaeon clone JMYA07_AC07 (82.55%)— FJ810526.1	**+**						
		no close match > 80%							
A13	MZ314660	*Haloarcula* sp. TG1 (96%)—KU051670.1	**+**	**+**		**+**	**+**		
A14	MZ314661	*Haloarcula* sp. HMC-3 (97%)—JX188260.1				**+**			
A15	MZ314662	*Haloarcula* sp. SS13-14 (99%)- KJ917665.1				**+**			
A16	MZ314663	Uncultured archaeon clone GSP_arch161 (96%)—FJ696332.1	**+**						
		*Haloarcula* sp. TG1 (96%)—KU051670.1							
A17	MZ314664	*Halorubrum* sp. YC16 (94%) – JN216859.1	**+**						
A18	MZ314665	Uncultured archaeon clone ss_030g (98.36%)—AJ969818.1	**+**						
		*Halanaeroarchaeum sulfurireducens* strain HSR2(97.42%)—CP008874.1							
A19	MZ314666	*Haloarcula* sp. ARA4_D05(98%)—KC313023.1				**+**			**+**
A20	MZ314667	Uncultured archaeon clone 3-07A (95.46%)—EF459703.1				**+**			
		Uncultured *Halorhabdus* sp. clone A2_31 (94.44%)—KM587767.1							

*The closed match and the closest identified type strain (in the case of unknown organisms) are indicated. All accession numbers correspond to GenBank except those for A1, A2, A3, and A12 that were deposited in NCBI Short Read Archive.*

**TABLE 3B:** 16S rRNA gene sequences detected in cultures developed on either acetate of lactate as the electron donor and fumarate as the electron acceptor upon amplification, DGGE separation, reamplification, and sequencing of bacterial DNA.

Operational Taxonomic Unit	Accession no.	Closest match (identity%)—accession no.	Fumarate	
			
		Closest identified type strain (identity%)—accession no.	Acetate	Lactate
B1	MZ314668	Uncultured bacterium clone HTA-6 (99%)—KY264962.1	**+**	**+**
		*Halanaerobium* sp. strain L4 (97.98%)—KX784553.1		
B2	MZ314669	Uncultured bacterium partial 16S rRNA gene, clone PB_72 (96%)—LN865077.1	**+**	
		no close match > 80%		
B3	SAMN22977629	*Halanaerobaculum tunisiense* strain 6SANG (96%)—NR_044464.1		**+**
B4	MZ314670	*Halanaerobium saccharolyticum* strain CEJFT1C (99%)—KU180223.1	**+**	**+**
B5	MZ314671	Uncultured bacterium clone W4 × 7_2B-9 (99%)—KF716151.1		**+**
		*Acetohalobium arabaticum* DSM 5501 (97.70)—NR_074672.1		
B6	MZ314672	*Sporohalobacter lortetii* strain CEJFYE2D (96%)—KU180227.1		**+**
B7	MZ314673	*Halanaerobium saccharolyticum* strain CEJFYE1A (100%)—KU180224.1		**+**

*The closed match and the closest identified type strain (in the case of unknown organisms) are indicated. Accession numbers are included.*

The composition of enriched communities will be analyzed from here on in relation to electron donor and acceptors utilization.

### Carbon Utilization

Under oxic conditions, lactate oxidation to pyruvate in bacteria is typically performed by membrane-associated NAD^+^-independent lactate dehydrogenases (iLDHs) that use membrane integral electron acceptors as, for example, ubiquinone in *Escherichia coli* (Anraku and Gennis, 1987) to drive electrons into the electron transport chain. Under anoxic conditions, on the other hand, lactate is often oxidized by soluble enzymes through recently described flavin-dependent electron bifurcation mechanisms (Buckel and Thauer, 2013) at expenses of, for example, the concomitant oxidation of reduced ferredoxin in acetogenic bacteria (Weghoff et al., 2015). Neither of these groups of proteins nor any protein with significant similarity to them is encoded in the *Halorubrum* and *Haloarcula* available genomes; however, all of them code for an LDH hypothetical protein (Table 4A) (LldG; e.g., DOS48_07575 in *Halorubrum* sp. PV6), which contains the recently described lactate utilization domain (LUD) (Hwang et al., 2013). In all these genomes, this gene is clustered with one coding for a ferredoxin-like protein of unknown function (e.g.,: DOS48_07580 in *Halorubrum* sp. PV6) that, in addition to the LUD domain, contains multiple 4Fe-4S binding domains (Table 4A). Both catalytic enzymes seem to be soluble proteins that are indeed structurally similar to members of the LldDEFG LDH complex described in *Shewanella oneidensis* MR1 (Supplementary Figure 4), with demonstrated functionality under both aerobic and anaerobic conditions (Pinchuk et al., 2009). This genomic information thus gives support to the enrichment of *Halorubrum* (Sequences A5, A6, A8, A9, and A11) and *Haloarcula* (sequences A7, A10, A13, A14, A15, and A19) related organisms growing on lactate (Table 3).

**TABLE 4A:** Presence or absence of genes coding for the indicated enzymes related to the utilization of lactate or acetate in the genome of listed organisms.

Organism	Lactate			Acetate

	nLDH	LldG	fdx-like LdH	A-CoA Syn

	1.1.1.27			6.2.1.1.
*Halorubrum* sp. PV6 (T05770)	−	+	+	+
*Halorubrum lacusprofundi* ATCC 49239 (T00856)	−	+	+	+
*Halorubrum sp. BOL3-1* (T05834)	−	+	+	+
*Halorubrum ezzemoulense strain Fb21* (T06071)	−	+	+	+
*Halorubrum CBA1229* (T06658)	−	+	+	+
*Haloarcula* sp. *CBA1115* (T03645)	−	+	+	+
*Haloarcula hispanica N601* (*T02949*)	−	+	+	+
*Haloarcula taiwanensis* (T05292)	−	+	+	+
*Haloarcula marismortui* ATCC 43049 (T00211)	−	+	+	+
*Haloarcula* sp. JP-L23 (T06489)	−	+	+	+
*Haloarcula hispanica* ATCC 33960 (T01597)	−	+	+	+
*Haloarculaceae archaeon HArcel1* (T05406)	−	+	+	+
*Halorhabdus utahensis DSM 12940* (T00971)	+	+	+	+
*Halorhabdus tiamatea SARL4B* (T02781)	+	–	–	+
*Halorhabdus* sp. *CBA1104* (T06289)	+	+	+	+
*Halanaeroarchaeum sulfurireducens* HSR2 (T03927)	−	−	−	+
*Halanaeroarchaeum sulfurireducens* M27-SA2 (T04261)	−	−	−	+
*Candidatus* Nanopetraeus SG9 (T04498)	1.1.1.28	−	−	+
*Halanaerobium praevalens* (T01941)	+	−	−	−
*Halanaerobium hydrogeniformans* (T01349)	+	−	−	−
*Halanaerobaculum tunisiense*	No genome available			
*Sporohalobacter lortetii*	No genome available			
*Acetohalobium arabaticum* (T01290)	+	−	−	−

*nLDH, lactate dehydrogenase; Lldg, hypothetical protein with a LUD (lactate utilization) domain; fdx-like LdH, ferredoxin-like hypothetical protein with an LUD domain and multiple 4Fe-4S binding domains; A-CoA Syn, acetyl CoA synthetase. Data were taken from KEGG database; accession numbers are included within parenthesis for reference.*

Upon lactate oxidation, pyruvate is converted to acetyl-CoA by pyruvate–ferredoxin oxidoreductases (Kerscher and Oesterhelt, 1981) and subsequently fed into the tricarboxylic acid (TCA) cycle. Alternatively, in some organisms, acetyl-CoA is not fed to the TCA cycle but is oxidized through the carbon monoxide dehydrogenase pathway (Thauer et al., 1977). In incomplete oxidizers, acetyl-CoA can yield acetate *via* acetyl-phosphate, which can generate ATP by substrate level phosphorylation (Thauer et al., 1977). Thus, acetate accumulates in the culture media or can be consumed by acetate-oxidizing microorganisms. Acetate oxidation can proceed through the action of the acetyl-CoA synthetase (EC 6.2.1.1) or can be activated to acetyl-CoA by acetate kinase (EC 2.7.2.1) and phosphotransacetylase (EC 2.3.1.8) (Schmitz et al., 2006). As indicated in Table 3, sequences A16 and A17, related to *Haloarcula* sp. TG1 (96%) and *Halorubrum* sp. YC16 (94%), respectively, were obtained from acetate fed cultures, in line with the presence of the gene encoding acetyl-CoA synthetase in all sequenced *Halorubrum* and *Haloarcula* genomes (Table 4A).

Sequence A18 was obtained from acetate-fed cultures using fumarate as the electron acceptor and matched with *H. sulfurireducens* (Table 3). *Halanaeroarchaeum* is a novel genus within the Halobacteriaceae whose cultivated members are obligate and strictly anaerobic organisms using acetate as the sole carbon and energy source and elemental sulfur as the electron acceptor (Messina et al., 2016). Accordingly, the two available genomes of *Halanaeroarchaeum* genus code for the ATP-dependent acetyl-CoA synthetase (EC 6.2.1.1) (Messina et al., 2016; Table 4A).

Sequence A20 was obtained from a lactate-fed culture and matched with *Halorhabdus* sp. clone A231 (94.44%). Members of the *Halorhabdus* genus were previously shown not utilizing lactate (Wainø et al., 2000; Antunes et al., 2008), but the three available genomes of this genus include the genes for an NAD^+^ dependent LDH (EC 1.1.1.27) and a racemase (EC 5.1.2.1). Both the genome of *Hrd. utahensis* DSM 12940 and that of *Halorhabdus* sp. CBA1104 additionally code for the LldG complex in association with the (4Fe-4S)–binding protein, as found in *Halorubrum* and *Haloarcula* representatives (Table 4A), which gives genetic support to the enrichment made here.

Sequences A1, A2, and A3 (Table 4) roughly matched an uncultured *Nanohaloarchaeota* member (89.46, 93.62, and 91.82%, respectively) found previously in water in the same site (Di Meglio et al., 2016) and also with *Candidatus* Nanopetraeus *SG9* (88%) (Table 3), both members of the class Nanohaloarchaea. This class is composed of very small-sized organisms grouped in the DPANN superphylum known as unexplored microbial dark matter (Rinke et al., 2013). They are ubiquitous inhabitants of different hypersaline environments all around the world (Jiang et al., 2007; Ghai et al., 2011; Narasingarao et al., 2011; Podell et al., 2013; Rinke et al., 2013; Gomariz et al., 2015; Ventosa et al., 2015; Di Meglio et al., 2016; Rubin et al., 2017), including saline soils (Vera-Gargallo and Ventosa, 2018; Gómez et al., 2019; Martínez et al., 2021). Despite this worldwide distribution, only *Candidatus Nanohaloarchaeum antarcticus*, an aerotolerant member of the Nanohaloarchaea from Rauer 1 Lake and Club Lake in Antarctica, could be successfully reproduced in laboratory conditions to present days (Hamm et al., 2019). Reproduction was in symbiotic association with *Halorubrum lacusprofundi* in line with the fact that metagenome-assembled genomes of *Nanohaloarchaeota* indicate they have evolved as symbionts (Hamm et al., 2019). Metabolic capabilities of these symbionts are very limited, but the genome of *Candidatus* Nanopetraeus SG9 contains a gene coding for an NAD^+^-dependent LDH (EC 1.1.1.28) (Table 4A) and also one coding for an acetyl-CoA ligase (EC 6.2.1.13). Band A1 was obtained from cultures grown on both acetate and lactate, whereas bands A2 and A3 were obtained from cultures grown on acetate, evidencing some metabolic difference within these organisms. The results of BLAST analysis partly support this difference by showing that A2 and A3 are 91% homologous, but share only 75 and 83% of their sequences with band A1, respectively, suggesting they all belong to different organisms.

Within enriched bacteria, band B5 was obtained from cultures grown on lactate and has *A. arabaticum* as the closely related (97.7%) known organism (Table 3). *A. arabaticum* is a strictly anaerobic fermentative bacteria capable of growing on lactate as the sole source of carbon (Sikorski et al., 2010; Kivistö and Karp, 2011); accordingly, coded in the genome of *A. arabaticum* are an LDH (Table 4) and a racemase (EC 5.1.2.1).

Three bacterial bands (B1, B4, and B7) matched with *Halanaerobium* strains, whereas the other two (B3 and B6) matched with *Halanaerobaculum* and *Sporohalobacter* strains, respectively (Table 3). These three genera are known to be composed by organisms that have been shown as unable to grow on lactate, neither on acetate (Hedi et al., 2009; Ben Abdallah et al., 2015; Oren, 2015), opening the question about how they could be enriched here. Unfortunately, there is no available genomic information from *Halanaerobaculum* and *Sporohalobacter* representatives. Available genomes of *Halanaerobium praevalens* and *Halanaerobium hydrogeniformans*, on the other hand, code for the NAD^+^ dependent LDH (EC 1.1.1.27) and for acetate kinase (EC 2.7.2.1) and phosphotransacetylase (EC 2.3.1.8), thus supporting the results presented here by indicating that these organisms may have the capability to utilize both lactate and acetate.

### Electron Acceptor Utilization

Although new reports have been emerging in recent times, available information on growth of hyperhalophiles performing anaerobic respiration is still limited. Despite this, nitrate, sulfate, thiosulfate, S^0^, fumarate, DMSO, TMAO, and even arsenate and selenate have been shown to support growth at high salt concentrations (Mancinelli and Hochstein, 1986; Oren and Trüper, 1990; Sorokin et al., 2017). The approach to obtain this information has been almost exclusively, attempting to grow every organism in axenic culture on the selected electron acceptor with a variety of electron donors. Here, a different approach has been used, aiming at enriching consortia in which ecological associations can maximize the possibilities of successful growth.

#### Fumarate Respiration

Fumarate respiration proceeds *via* reduction to succinate by the fumarate reductase, which catalyzes the reverse reaction of succinate dehydrogenase (Kröger et al., 1992; Iverson, 1999). In this work, several archaea related to *Halorubrum*, *Haloarcula*, *Halanaeroarchaeum*, and *Halorhabdus* genera and also bacteria related to *Halanaerobium*, *Halanaerobaculum*, *Sporohalobacter*, and *Acetohalobium* have been enriched in cultures using fumarate as the only available electron acceptor (Table 3). Fumarate reductase (EC 1.3.5.4) is coded in all available genomes belonging to archaea genera related to enriched organisms (Table 4B), but growth of haloarchaea using fumarate as the terminal electron acceptor was previously reported only in *H. salinarum*, *Haloferax volcanii*, and *H. denitrificans*, and this is, to our knowledge, the first time that representatives of *Haloarcula*, *Halorubrum*, *Halanaeroarchaeum*, and *Halorhabdus* are repeatedly grown on this type of respiration.

**TABLE 4B:** Presence or absence of genes coding for the indicated enzymes related to the utilization of the indicated electron acceptor in the genome of listed organisms.

Organism	Fumarate	Nitrate					Sulfate			S_0_/polysulfide	DMSO

	Fum red	Nitrate reductase NarGHI/NapAB	Nitrite reductase NirK/NirS (NO)	Nitrite reductase NirA (NH4 +)	Nitric oxide reductase NorBC	Nitrous oxide reductase NosZ	ATP sulfurylase	APS reductase	Sulfite reductase	Polysulfide reductase NrfD	Dimethylsulfoxide reductase

	1.3.5.4	1.7.5.1	1.7.2.1	1.7.7.1	1.7.2.5	1.7.2.4	2.7.7.4	1.8.99.2	1.8.99.1		1.8.5.3
*Halorubrum* sp. *PV6*	+	−	−	+	−	−	−	−	−	−	−
*Halorubrum lacusprofundi* ATCC 49239	+	+	−	+	+	+	−	−	−	−	+
*Halorubrum* sp. BOL3-1	+	−	−	−	−	−	−	−	−	+	−
*Halorubrum ezzemoulense* strain Fb21	+	−	−	−	−	−	−	−	−	−	−
*Halorubrum* CBA1229	+	+	−	+	+	+	−	−	−	+	+
*Haloarcula* sp. CBA1115	+	+	+	+	+	+	−	−	−	−	+
*Haloarcula hispanica* N601	+	+	+	+	+	+	−	−	−	−	+
*Haloarcula taiwanensis* strain *Taiwanensis*	+	+	+	+	+	+	−	−	−	−	+
*Haloarcula marismortui* ATCC 43049	+	+	+	+	+	+	−	−	−	−	−
*Haloarcula* sp. JP-L23	+	+	+	+	+	+	−	−	−	−	+
*Haloarcula hispanica* ATCC 33960	+	+	+	+	+	+	−	−	−	−	+
*Haloarculaceae archaeon* HArcel1	+	−	−	+	−	−	−	−	−	−	−
*Halorhabdus utahensis* DSM 12940	+	+	+	+	−	−	−	−	−	−	+
*Halorhabdus tiamatea* SARL4B	+	−	−	+	−	−	−	−	−	−	−
*Halorhabdus* sp. CBA1104	+	+	+	+	−	−	−	−	−	−	+
*Halanaeroarchaeum sulfurireducens* HSR2	+	−	−	−	−	−	−	−	−	+	−
*Halanaeroarchaeum sulfurireducens* M27-SA2	+	−	−	−	−	−	−	−	−	−	−
*Candidatus* Nanopetraeus SG9	−	−	−	−	−	−	−	−	−	−	−
*Halanaerobium praevalens*	+	−	−	−	−	−	−	−	−	−	−
*Halanaerobium hydrogeniformans*	−	−	−	−	−	−	−	−	−	−	−
*Halanaerobaculum tunisiense*	No genome available
*Sporohalobacter lortetii*	No genome available
*Acetohalobium arabaticum*	−	−	−	−	−	−	−	−	+	−	−

The finding of organisms with close similarity to Nanohaloarchaea members in cultures grown on fumarate as the electron acceptor (Table 3A) deserves particular consideration. As shown in Table 4B, the genome of *Candidatus* Nanopetraeus SG9 evidences the complete lack of genes coding for main reductases involved in nitrate, fumarate, DMSO, and sulfate respiration processes, suggesting that this organism gains ATP from its host, as suggested also for *Nanoarchaeum equitans*, a symbiont of the hyperthermophilic, strictly anaerobic crenarchaeon *Ignicoccus hospitalis* (Mayer and Müller, 2014), and for *Candidatus Nanopusillus acidilobi*, a nanosized exosymbiont of the thermoacidophilic anaerobe *Acidilobus* sp. 7A (Wurch et al., 2016). Taking into account the association of *Candidatus N. antarcticus* with *H. lacusprofundi*, it is hypothesized that putative Nanohaloarchaea reproduced here in cultures grown anaerobically on fumarate would gain ATP from the symbiotic association, with some of the various *Halorubrum* representatives enriched in the same consortia (Table 3A).

Regarding enriched bacteria, fumarate respiration has not been reported previously in any of the four genera enriched here. As shown in Table 4B, none of the genomes belonging to identified genera include the gene for fumarate reductase (E.C. 1.3.5.4), but the genomes of both *H. praevalens* and *A. arabaticum* code for a protein with fumarate reductase/succinate dehydrogenase activity that may be operative in reducing fumarate to succinate (Hprae_0865 and Acear_1952, respectively) for producing ATP, as described, for example, in *Propionibacterium* (Kim and Gadd, 2019). In relation to this, it is interesting to note that along cultivation of *H. volcanii* on fumarate as an oxidizer (Oren, 2011), succinate was accumulated in the culture media. If this was the case here with fumarate respiration, assimilatory succinate utilization by other community member(s) could explain extended growth as that reported in Supplementary Figure 1 and the better GP on fumarate as compared with that on DMSO and thiosulfate (Figure 2). Partly supporting this possibility, succinate membrane transporters have been detected in various halophilic microorganisms, as, for example, *Halobacterium*, *Haloarcula*, and *Halococcus* strains (Plakunov et al., 1984), whereas succinate has been proven to be used as carbon source by *H. volcanii*, *Halobacterium saccharovorum*, *Haloarcula vallismortis*, and other hyperhalophiles (Kauri et al., 1990). It is important to note at this time that, although growth on fumarate was to a higher OD_600_, it was slower than that on thiosulfate and DMSO (Table 2), suggesting the occurrence of slow steps in energy utilization, as could be expected upon the use of secondary metabolites at the community level.

#### Nitrate Respiration

Nitrate respiration is an anaerobic process whereby nitrate is sequentially reduced to nitrite, NO, N_2_O, and N_2_ by nitrate reductase NarGHI, nitrite reductase NirK/NirS, nitric oxide reductase NorBC, and nitrous oxide reductase NosZ, respectively. Nitrate is rarely found at high concentrations in hypersaline environments, and to date, the capability to grow on nitrate respiration has been experimentally probed only in *H. marismortui*, *H. vallismortis*, *H. denitrificans*, *Haloferax mediterranei*, and *Halogeometricum borinquense* (Mancinelli and Hochstein, 1986; Montalvo-Rodriguez et al., 1998; Bonete et al., 2008; Torregrosa-Crespo et al., 2020). Several bands were obtained in the DGGE analyses performed on nitrate-respiring cultures (Supplementary Figure 3), but only four could be amplified, sequenced, and identified. Two of them (A7 and A19) were related to *Haloarcula* representatives, whereas the other two (A8 and A9) matched with *Halorubrum* ones. Six of seven available genomes of *Haloarcula* code for the complete set of enzymes for reducing nitrate to N_2_ (Table 4B), including NarG and NarH homologs of the Nar complex, the copper-containing variant of nitrite reductase (NirK), and the NorB and NosZ homologs, which give support to experimental results. Within *Halorubrum* genomes on the other side, only *H. lacusprofundi* ATCC 49239 and *Halorubrum* sp. CBA1229 have some of the denitrifying enzymes, but present an incomplete pathway because of the absence of NirK (Table 4B). All *Halorubrum* strains are in consequence impeded to grow on nitrate reduction in axenic cultures, a fact that has been repeatedly proved along years (Castillo et al., 2007; Yim et al., 2014; Gibtan et al., 2018). This restriction can be clearly circumvented when growing in association with a nitrite-reducing organism, as is the case here with *Haloarcula* representatives (Tables 3, 4) that besides consuming nitrite have been demonstrated to accumulate N_2_O in the stationary phase (Bonete et al., 2008), thus providing *Halorubrum* with an additional acceptor for producing energy (Kim and Gadd, 2019).

#### Electrode Respiration

Respiration of polarized electrodes has emerged as a new process with practical implications in the production of bioelectricity, the production of bioelectrochemical sensors, and the bioelectrochemical remediation of contaminated waters and soils, within other technological developments (Rosenbaum and Franks, 2014). It is based on exocellular respiration, a capacity that evolved in bacteria to profit on the reduction of solid compounds present in the environment, as, for example, iron and manganese oxides or S_0_. In exocellular respiration, an extension of the internal electron transport chain reaches the cell exterior to contact redox molecules in the outermost cell surface, which finally interact to the solid electron acceptor. Cell external molecules involved in electrode respiration have been shown to be cytochromes of the c-type (Busalmen et al., 2008, 2010; Logan, 2009), some of which have also been related to iron oxide reduction (Butler et al., 2010). Exocellular respiration was initially found in a few *Geobacter* and *Shewanella* strains, but as the use of electrochemical enrichment techniques expanded, it was rapidly identified as a common feature in microbial anaerobic ecosystems (Koch and Harnisch, 2016). Although not allowing measurable growth (by OD_600_), electrochemical conditioning performed here led to the production of an increasing current (Figure 1), pointing to a progressive electric interaction of cells with the polarized electrode. As a result, several archaeal representatives were enriched, which were closely related to *Halorubrum* (bands A6, A9, and A11) and *Haloarcula* (band A11) strains (Table 3A). The electric interaction mechanism allowing enrichment is not known, but it may involve external redox molecules with a half-wave redox potential of approximately 0.0 V (Supplementary Figure 2), close to that of iron oxide reductases typically found in *Geobacter* (Marsili et al., 2010). Iron oxide reduction has not been reported in halophilic archaea. In place, there are archaea that reduce S_0_ (Liu et al., 2012; Messina et al., 2016; Kim and Gadd, 2019), a solid electron acceptor with a redox potential of −0.275 V (Thauer et al., 1977). This function is thought to be performed by membrane-bound reductases that would directly interact with S_0_ (Liu et al., 2012; Kim and Gadd, 2019), thus completing an exocellular respiration process. The genomes of *Halorubrum* BOL3-1 and *Halorubrum* CBA1229, as well as that of *H. marismortui*, containing different operons coding for molybdopterin oxidoreductases resembling those in *Halanarchaeum* (Messina et al., 2016), could be operative in exporting electrons to the cell outside, but additional research is needed to determine if they are involved in the electrochemical processes reported in Supplementary Figure 2.

#### Sulfate Respiration

Dissimilatory sulfate reduction is present in bacteria and archaea and is known to occur up to high salt concentration approaching saturation (Oren, 1999; Kjeldsen et al., 2007). In fact, black deposits were present in the sampling site of Salitral Negro (Supplementary Figure 5), where the sulfate content of water is known to seasonally vary between 8.6 and 23.6 g/L (Di Meglio et al., 2016), whereas the content in pore brine has been shown to reach 89 g/L (Pires, 2017). Despite having detected different archaea and bacteria in the DGGE analysis of consortia enriched on sulfate (Supplementary Figure 3), only four archaea bands could be amplified, sequenced, and identified. A6, A8, and A9 matched with *Halorubrum* representatives, whereas A13 matched with *Haloarcula* (Table 3A). According to available data, dissimilatory sulfate reduction is not widespread within archaea, but concentrated in a few genera including *Archaeoglobus* of the phylum Euryarchaeota and *Pyrobaculum*, *Thermocladium*, and *Caldivirga* of the phylum Crenarchaeota (Liu et al., 2012). With a few exceptions, available genomes of these genera contain the complete bacterial pathway for dissimilatory sulfate reduction including the sulfate adenylyltransferase (Sat) for activation of sulfate to form adenosine-5′-phosphosulfate (APS), the APS reductase (AprBA) that reduces APS to sulfite, and, finally, the dissimilatory sulfite reductase (DsrAB) that reduces sulfite to sulfide.

Growth observed here of *Halorubrum* and *Haloarcula* representatives on sulfate respiration is intriguing, as their genomic information indicates that none of sequenced species contain the genes coding for any of the elements of the bacterial sulfate reduction pathway (Table 4B). Taking into account that both *Halorubrum* and *Haloarcula* representatives were enriched also on electrodes (Table 3A), a hypothetical explanation to the unexpected growth of these organisms in sulfate respiring cultures is that they can perform direct interspecies electron transport (DIET) with sulfate reducing neighborhoods. DIET has been called to be a third mechanism of syntrophy (Walker et al., 2020) that adds to the generally accepted exchange of formate or H_2_. Similarly, it could be active here in channeling electrons to sulfate as the final electron acceptor. In this direction, cultures grown on electrodes can produce electrons at a potential as negative as −0.2 V (Supplementary Figure 2), which would be negative enough to allow a synthropy partner to gain energy upon using these electrons in sulfate reduction (Su et al., 2012).

#### Dimethyl Sulfoxide Respiration

Despite having enriched several bacteria and archaea in cultures grown on DMSO, only a single band belonging to an archaea could be reamplified, sequenced, and identified. It was band A13 (Table 3A), which presented a 96% similarity with *Haloarcula* sp. TG1. The ability to use DMSO for anaerobic respiration has been previously reported in different strains of *Haloarcula*, as well as in *Halobacterium*, *Haloferax* (Oren and Trüper, 1990), and more recently in the members of the newly proposed *Halodesulfurarchaeum* genus (Sorokin et al., 2017). As shown in Table 4B, five out of seven *Haloarcula* genomes available at KEGG database include a gene coding for a DMSO reductase.

## Conclusion

The microflora inhabiting the submerged sediment of Salitral Negro, La Pampa province, Argentina, was challenged to grow under anaerobic conditions. Growth was demonstrated on all offered electron acceptors, including fumarate, nitrate, sulfate, thiosulfate, DMSO, and even a polarized electrode, which evidences potential industrial applications. GP of enriched consortia was markedly high on nitrate, despite that this anion is virtually absent in this natural environment. On the contrary, although sulfate is known to be present at relatively high concentration, it does not support massive growth. The analysis performed on enriched consortia revealed that *Halorubrum* and *Haloarcula* representatives could be grown for the first time on lactate, indeed using fumarate or a polarized electrode as the electron acceptor. In addition, they were found composing sulfate-reducing consortia. *Halorubrum* representatives were also found composing nitrate-reducing consortia. Fumarate respiration was shown for the first time supporting growth of *Halanaeroarchaeum*- and *Halorhabdus*-belonging archaea, as well as of *Halanaerobium*-, *Halanaerobaculum*-, *Sporohalobacter*-, and *Acetohalobium*-belonging bacteria. Finally, evidence was presented suggesting growth of nanohaloarchaea in anaerobic conditions.

## Data Availability Statement

The datasets presented in this study can be found in online repositories. The names of the repository/repositories and accession number(s) can be found in the article/Supplementary Material.

## Author Contributions

JS performed the experiments. JB and DN designed the experiments, discussed the results, and wrote the manuscript. All authors contributed to the article and approved the submitted version.

## Conflict of Interest

The authors declare that the research was conducted in the absence of any commercial or financial relationships that could be construed as a potential conflict of interest.

## Publisher’s Note

All claims expressed in this article are solely those of the authors and do not necessarily represent those of their affiliated organizations, or those of the publisher, the editors and the reviewers. Any product that may be evaluated in this article, or claim that may be made by its manufacturer, is not guaranteed or endorsed by the publisher.

## Footnotes

1
http://www.ncbi.nlm.nih.gov/

2
https://www.genome.jp/kegg/
