# Supplementary material for: Unraveling Anaerobic Metabolisms in a Hypersaline Sediment
Source: Front Microbiol. 2022 Mar 16;13:811432. doi: 10.3389/fmicb.2022.811432 (PMC8966722; doi:10.3389/fmicb.2022.811432)
Supplement: Supplementary file 1 [file Data_Sheet_1.DOCX]

**SUPPLEMENTARY MATERIAL**

**Unraveling anaerobic metabolisms in hypersaline sediment**

Solchaga, Juan Ignacio^1^; Busalmen, Juan Pablo^2^ and Nercessian, Débora^1^.

^1^Instituto de Investigaciones Biológicas (CONICET-UNMdP). Funes 3250, (7600) Mar del Plata, Argentina.

^2^Laboratorio de Bioelectroquímica, INTEMA (CONICET-UNMdP). Av Colón 10850, (B7606BWV) Mar del Plata, Argentina.

A)

**
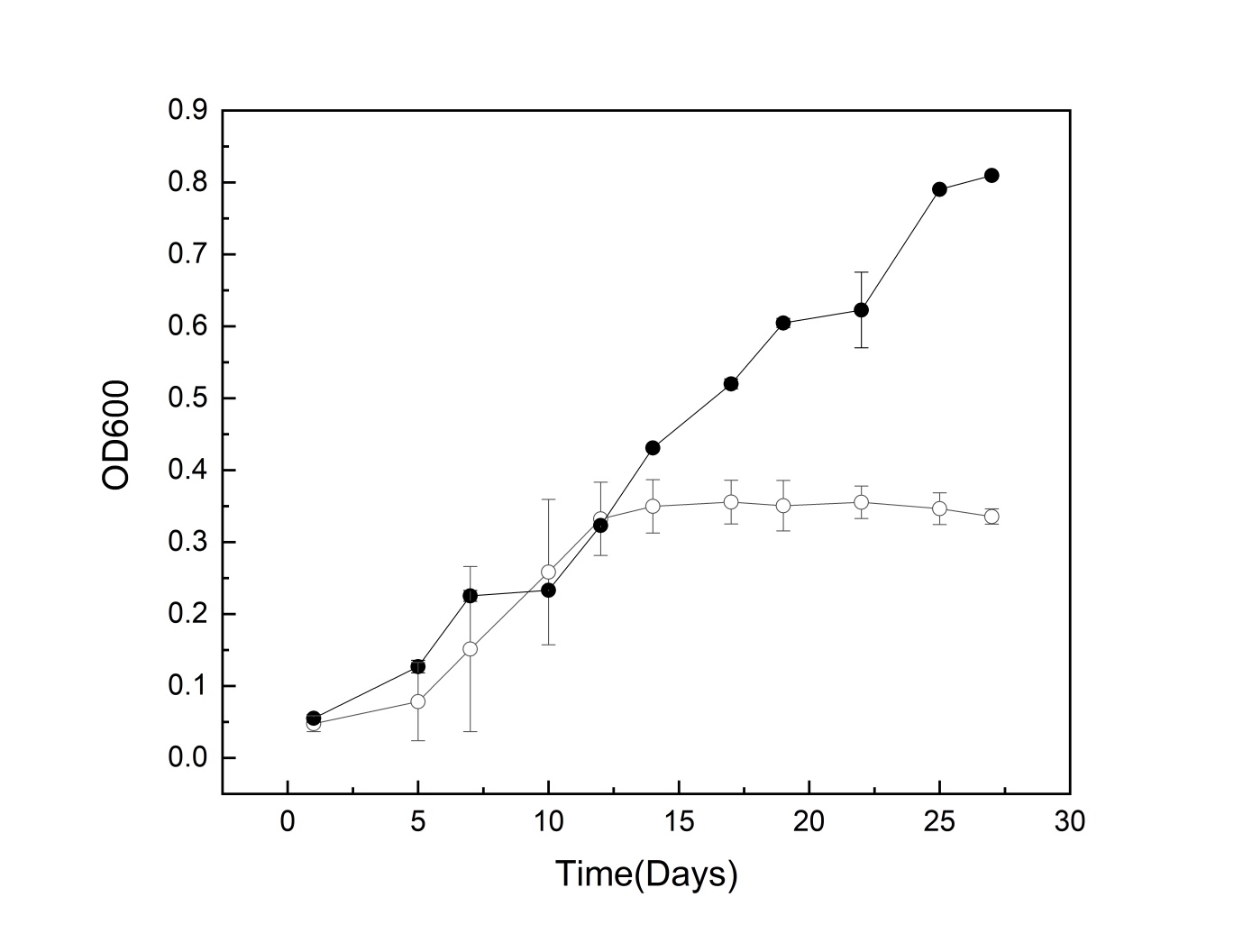
**

B)

**
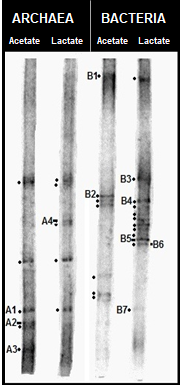
**

**Figure S1:** A) Evolution of growth of the autochthonous microflora of Salitral Negro on defined medium containing sodium acetate (○) or sodium lactate (●) as the electron donor and sodium fumarate as the electron acceptor. Data are presented as mean ± standard deviation (n≥3). B) Photograph showing typical results of DGGE analysis of bacterial and archaeal 16S rRNA gene fragments of communities growing on fumarate and the indicated reducer, amplified with bacterial (341F and 907R) and archaeal (344F and 907R) degenerate primers. Dots indicate the bands that were excised and amplified again for sequencing analysis. Bands belonging to organisms that could be identified are indicated with letters.

**
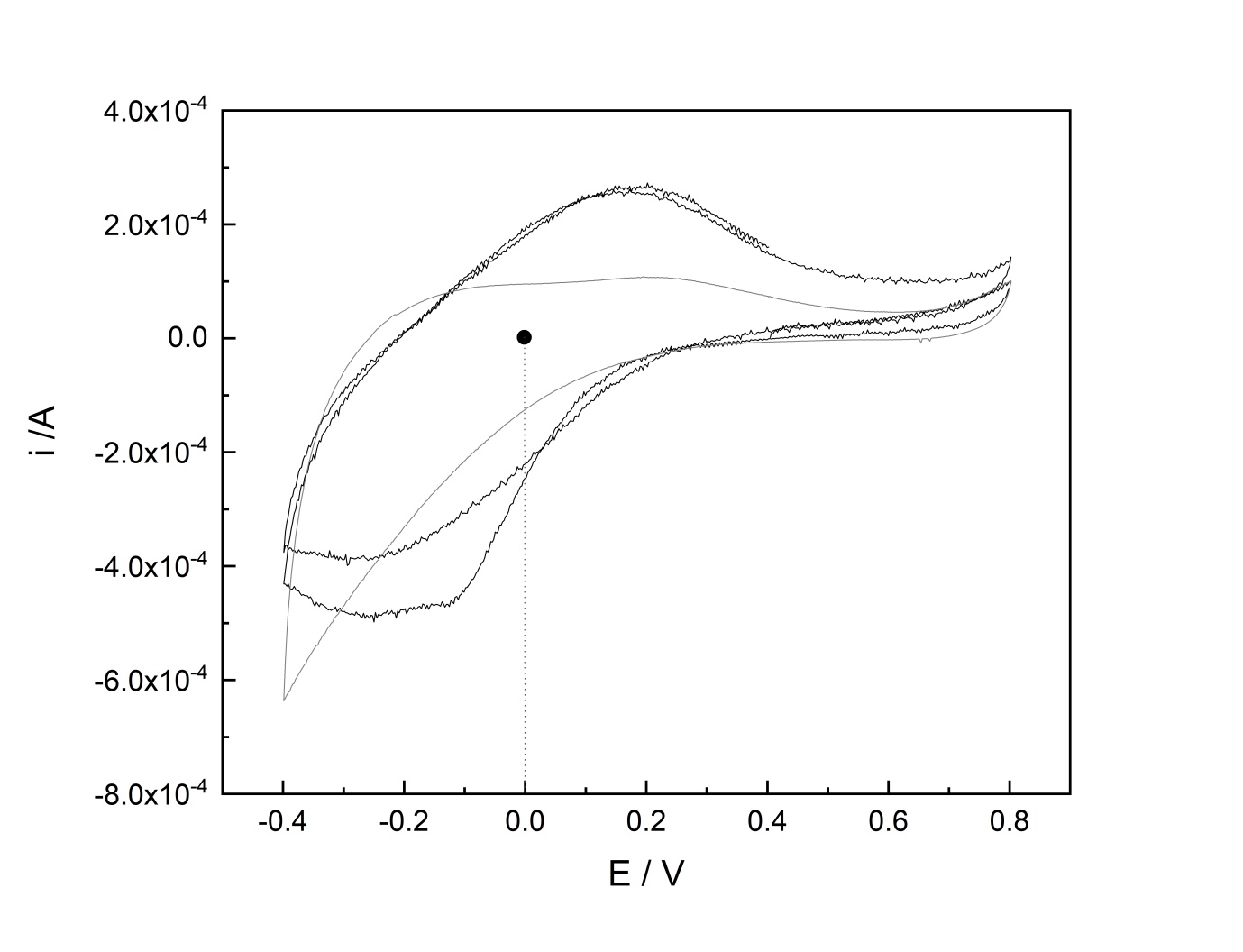
Figure S2:** a) Cyclic voltammetry obtained from a graphite electrode polarized to 0,4 V (SHE) and used as the only electron acceptor for anaerobic growth over 12 days (Fig. 1). Electrochemical reactor was inoculated with an enrichment of the original microflora of Salitral Negro, Argentina, using the lactate/acetate donor/acceptor couple as described in the text. The potential was sweep between -0,8 and +0,4 V starting anodically from the polarization potential at 0,01 V. s^-1^. The black spot indicates the half wave redox potential of the bacterial redox couple estimated as the intersection of the peak to peak straight line with ordinate axes. The response obtained from an electrode exposed to sterile control is included (grey line).

**
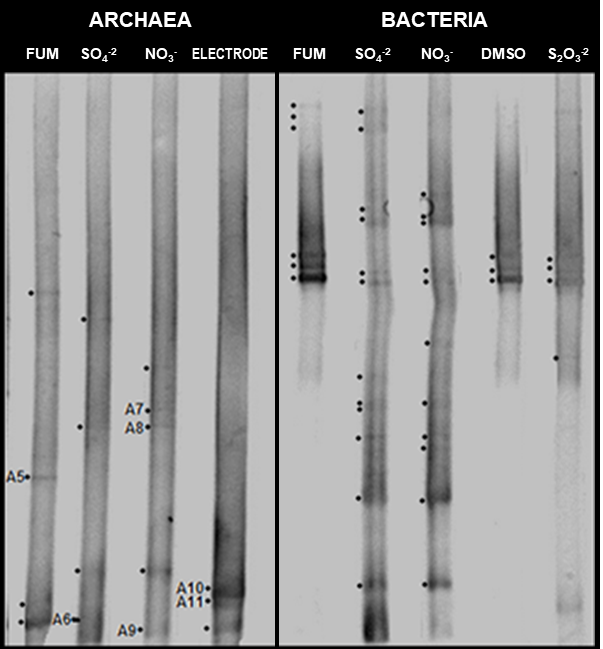
**

**Figure S3**: DGGE analysis of bacterial and archaeal 16S rRNA gene fragments obtained from microbial enrichments growing on lactate and the indicated oxidizers, amplified with bacterial (344F and 907R) and archaeal (341F and 907R) primers. Dots indicate the bands that were excised and amplified again for sequencing analysis. Bands belonging to organisms that could be identified in this particular assay are indicated with letters.


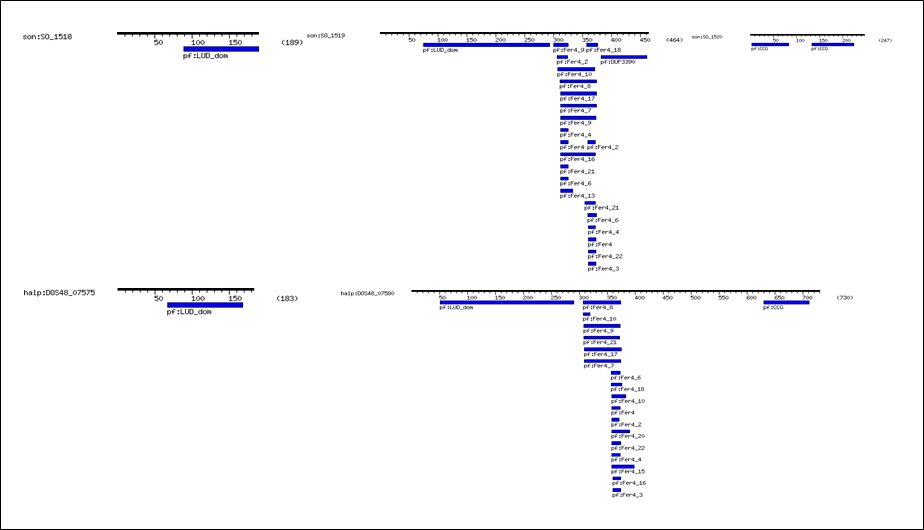


**Figure S4:** Structural similarity between the LldDEFG lactate dehydrogenase complex described in *Shewanella oneidensis* MR1 and the lactate dehydrogenase hypothetical protein cluster found in all *Halorubrum* and *Haloarcula* genomes available in KEGG database.


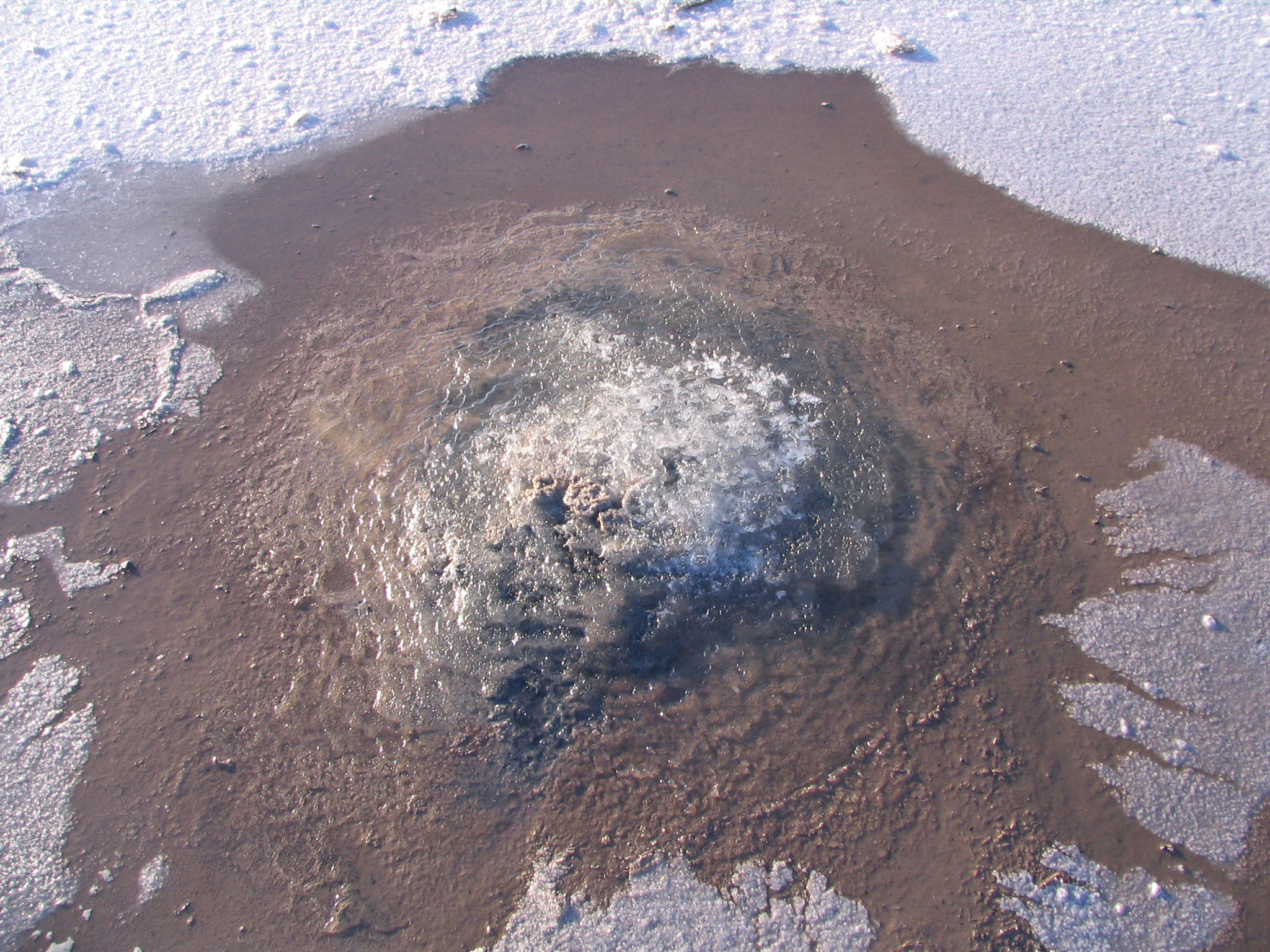


**Figure S5:** Photograph showing the occurrence of black deposits (indicated by arrows) in the sediments of Salitral Negro, Argentina.
